# Anisotropic Kernels for Coordinate-Based Meta-Analyses of Neuroimaging Studies

**DOI:** 10.3389/fpsyt.2014.00013

**Published:** 2014-02-10

**Authors:** Joaquim Radua, Katya Rubia, Erick Jorge Canales-Rodríguez, Edith Pomarol-Clotet, Paolo Fusar-Poli, David Mataix-Cols

**Affiliations:** ^1^Department of Psychosis Studies, Institute of Psychiatry, King’s College London, London, UK; ^2^Research Unit, FIDMAG Germanes Hospitalàries – CIBERSAM, Barcelona, Spain; ^3^Department of Child and Adolescent Psychiatry, Institute of Psychiatry, King’s College London, London, UK; ^4^Department of Clinical Neuroscience, Karolinska Institutet, Stockholm, Sweden

**Keywords:** activation likelihood estimation, anisotropic kernel, coordinate-based meta-analysis, effect size, magnetic resonance imaging, neuroimaging, signed differential mapping

## Abstract

Peak-based meta-analyses of neuroimaging studies create, for each study, a brain map of effect size or peak likelihood by convolving a kernel with each reported peak. A kernel is a small matrix applied in order that voxels surrounding the peak have a value similar to, but slightly lower than that of the peak. Current kernels are isotropic, i.e., the value of a voxel close to a peak only depends on the Euclidean distance between the voxel and the peak. However, such perfect spheres of effect size or likelihood around the peak are rather implausible: a voxel that correlates with the peak across individuals is more likely to be part of the cluster of significant activation or difference than voxels uncorrelated with the peak. This paper introduces anisotropic kernels, which assign different values to the different neighboring voxels based on the spatial correlation between them. They are specifically developed for effect-size signed differential mapping (ES-SDM), though might be easily implemented in other meta-analysis packages such as activation likelihood estimation (ALE). The paper also describes the creation of the required correlation templates for gray matter/BOLD response, white matter, cerebrospinal fluid, and fractional anisotropy. Finally, the new method is validated by quantifying the accuracy of the recreation of effect size maps from peak information. This empirical validation showed that the optimal degree of anisotropy and full-width at half-maximum (FWHM) might vary largely depending on the specific data meta-analyzed. However, it also showed that the recreation substantially improved and did not depend on the FWHM when full anisotropy was used. Based on these results, we recommend the use of fully anisotropic kernels in ES-SDM and ALE, unless optimal meta-analysis-specific parameters can be estimated based on the recreation of available statistical maps. The new method and templates are freely available at http://www.sdmproject.com/.

## Introduction

**Figure 1:**
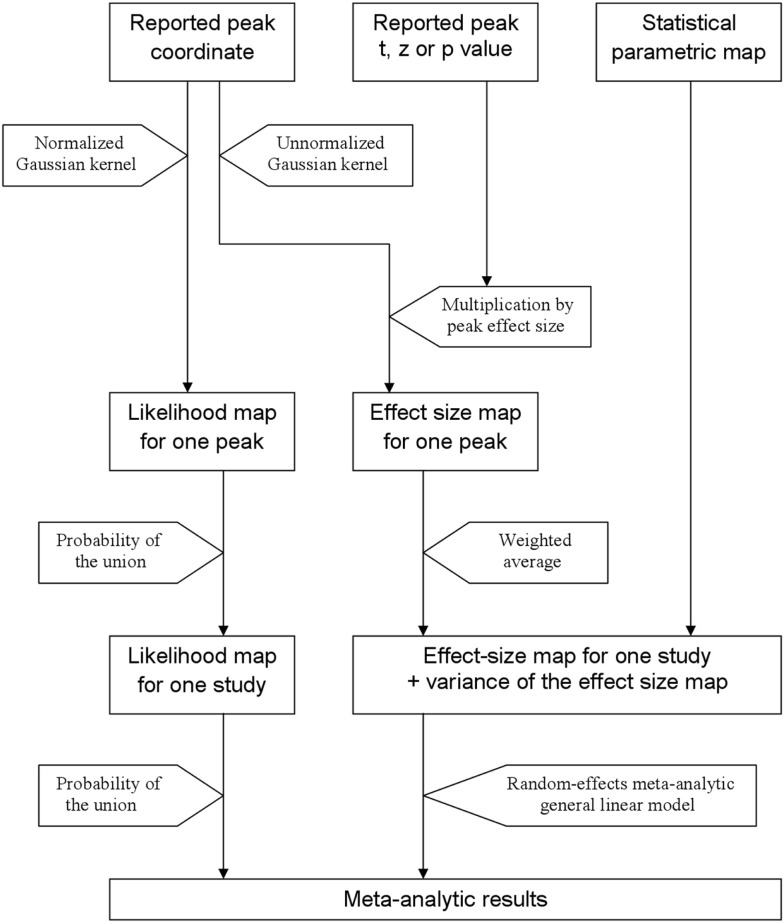
**Main steps of activation likelihood estimation (ALE) and effect-size signed differential mapping (ES-SDM)**. ALE (left approach) aims to estimate the likelihood that a peak lies in any given voxel. To this end, it first applies a Gaussian kernel so that the likelihood is high in the voxel where the peak is reported and similar but slightly lower in the close voxels. Afterward, it calculates the probability of the union of the likelihoods estimated from the different peaks and studies. ES-SDM algorithms (middle and right approach) are different, as this method aims to estimate the effect size rather than the peak likelihood. However, the first step also consists in applying an (un-normalized) Gaussian kernel, this time to achieve that voxels around a reported peak have an estimated effect size which is similar but slightly smaller to that of the peak. Afterward, effect-sizes recreated from the different peaks of a study are combined using a weighted average, i.e., when a voxel is close to two peaks, it has an effect size that depends on both peaks. Finally, the effect size maps as well as their variance maps are introduced in a meta-analytic random-effects general linear model.

**Figure 2:**
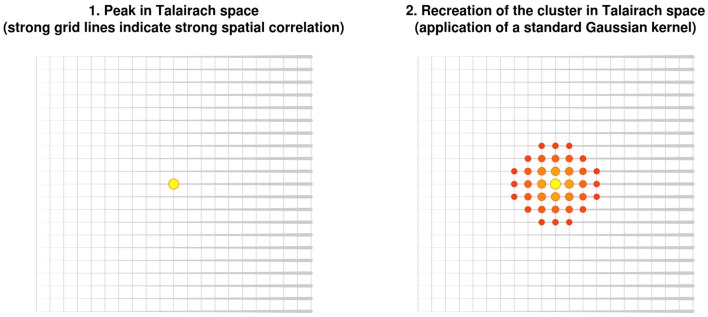
**Recreation of clusters using isotropic kernels in previous versions of effect-size signed differential mapping (ES-SDM) and activation likelihood estimation (ALE)**. Note that the recreation of the effect size (or the estimation of the activation likelihood) does *not* depend on the strength of the spatial correlations, but only on the Euclidean distance between each voxel and the peak.

In order to help summarize and integrate the results of the ever-growing number of neuroimaging studies, some groups have developed methods to conduct voxel-based meta-analyses solely relying on the information reported in the papers, namely the peaks of the clusters where there were statistically significant activations or where patients and controls showed statistically significant differences. Activation likelihood estimation (ALE) (1–4), (effect-size) signed differential mapping (ES-SDM) (5–7) and (multilevel) kernel density analysis (M-KDA) (8, 9) are commonly used methods that have already been applied to meta-analyze a wide range of normal brain functions (10–12) and abnormalities in neurological (13–15) and psychiatric disorders (16–18). As briefly introduced in Figure 1, these methods differ substantially in their algorithms [for a deeper review, see Ref. (19)], but one characteristic they share is that all convolve an isotropic kernel with the peak. In this context, a “kernel” is a small matrix convolved with the peaks in order that voxels surrounding a peak have a value similar to, but slightly lower than that of the peak. An “isotropic kernel” is identical in all directions. In simple terms, the effect size of a voxel close to a peak would only depend on the effect size of the peak and the Euclidian distance between the voxel and the peak (Figure 2). All voxels at 1 cm of a peak would have the same effect size, independently of whether they are in the same brain region or not.

However, such perfect spheres of effect size around the peak are probably implausible. Independently on the distance, voxels in the same brain region as the peak are more likely to be part of the cluster of significant activation or difference. Conversely, voxels in other brain regions, or separated from the peak by cerebrospinal fluid, are less likely to be part of the cluster.

Applying an isotropic kernel may thus underestimate the effect size of voxels in the same brain region as the peak, whereas it may overestimate the effect size of voxels from other brain regions. Some groups have recommended the use of large kernels, thus minimizing the underestimation of the effect size, though at the cost of a potential overestimation in some voxels (6, 20). Other developers have recommended the use of narrow kernels, thus minimizing the overestimation of the effect size, though at the cost of potential underestimation in some other voxels (4).

The aim of this study was to develop anisotropic kernels for coordinate-based meta-analyses, which would assign different values to the different neighboring voxels based on the spatial correlation between them. This was specifically developed for ES-SDM, although it might be easily implemented in other widely used meta-analytical programs such as ALE. The paper also includes the creation of new tissue-specific templates and a validation of the new method. We hypothesized that the recreation of effect size maps using anisotropic kernels would be more accurate than using isotropic kernels.

## Theory

Previous versions of ES-SDM adopted the ALE Gaussian kernel with the aim that, in the recreated statistical map, the voxels close to a peak have slightly smaller effect sizes than that of the peak, and progressively further voxels have progressively smaller effect sizes. Specifically, the effect size of a voxel close to a peak depended on the effect size of the peak and on the Euclidean distance between the voxel and the peak by means of an un-normalized Gaussian function:

(1) $$d_{\mathrm{voxel}}=\exp\left(\frac{-D^{2}}{2\cdot \sigma^{2}}\right)\cdot d_{\mathrm{peak}}$$

where *d* is the effect size, *D* is the distance, and σ is the standard deviation of the kernel (approximately 0.425 of its full-width at half-maximum, FWHM).

**Figure 3:**
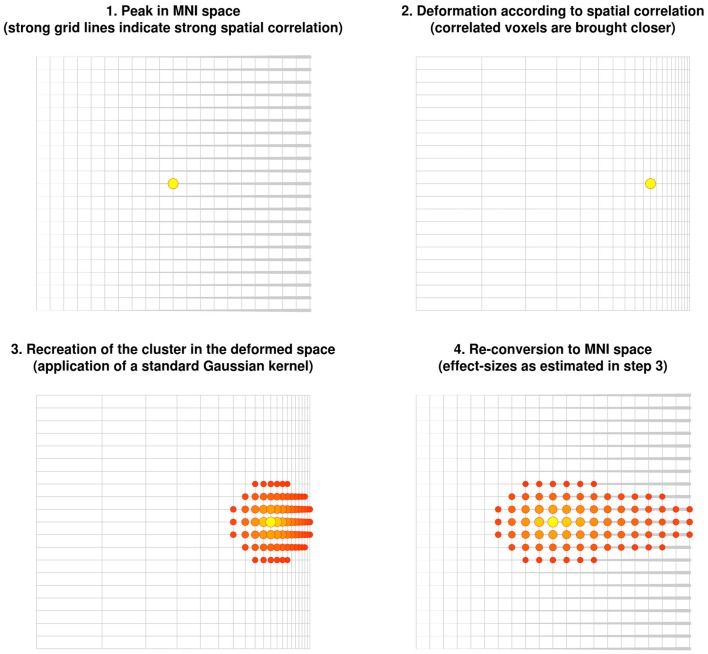
**Recreation of clusters using the anisotropic kernel in the updated version of effect-size signed differential mapping (ES-SDM)**. Note that the recreation of the effect size *does* depend on the strength of the spatial correlations, with the cluster being stretched toward voxels highly correlated with the peak.

The new method described here is based on the correlation between close voxels in the underlying structural image. Note that correlated voxels (e.g., individuals with much gray matter in one voxel tend to also have much gray matter in the other voxel) are more likely to be from the same brain region. The method consists of virtually deforming the distance so that highly correlated voxels are brought closer, while uncorrelated voxels are moved further away. A Gaussian kernel is then applied to the deformed space. When the original space is restored, highly correlated voxels are estimated to have larger effect sizes whereas uncorrelated voxels are estimated to have smaller or null effect sizes (Figure 3).

### Deformation of the space

Space is deformed to match the correlation of each voxel with its neighbors. To match distances and correlations, the expression of *d*_voxel_ in Eq. 1 is made equal the expression of *d*_voxel_ in Eq. 2, obtaining Eq. 3 from which *D* may be isolated:

(2) $$\begin{array}{ll} & d_{\mathrm{voxel}}=\rho \cdot d_{\mathrm{peak}} \end{array}$$

(3) $$\begin{array}{ll} & \exp\left(\frac{-D^{2}}{2\cdot \sigma^{2}}\right)\cdot d_{\mathrm{peak}}=\rho \cdot d_{\mathrm{peak}} \end{array}$$

(4) $$\begin{array}{ll} & D=\sqrt{2\cdot \sigma^{2}\cdot \log\left(\rho^{-1}\right)} \end{array}$$

where ρ is the coefficient of correlation between the voxel and the peak.

**Figure 4:**
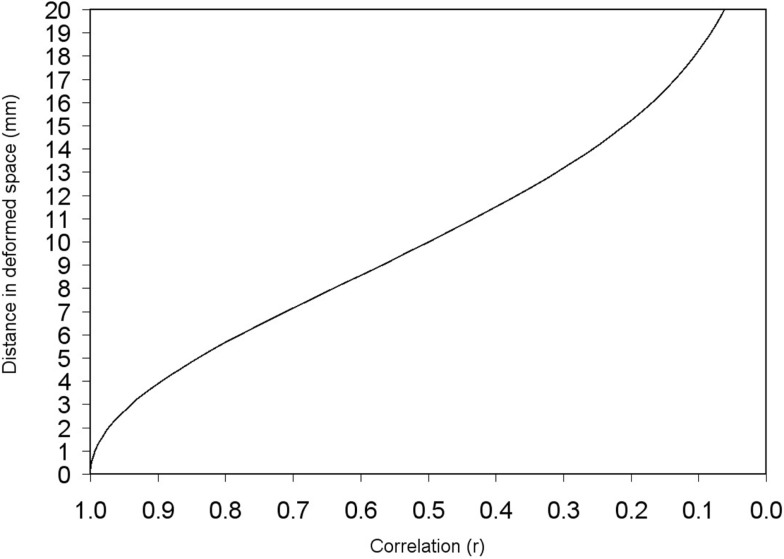
**Deformed distance between two adjacent voxels depending on the correlation between them**. Deformed distances in this example have been calculated for σ = 8.5 mm (FWHM = 20 mm). Note, however, that the recreation of effect size map does not indeed depend on FWHM when full anisotropy is used [see text, Eq. 2 and Figure 6].

The distance between a peak and its adjacent voxels is thus deformed according to the Eq. 4, the only variables of which are the constant standard deviation of the kernel (or equivalently the FWHM) and the correlation between the two voxels. Figure 4 shows this correspondence between correlation and deformed space.

To estimate the distance between the peak and a non-contiguous voxel *v*, the software must sum the distances between the pairs of contiguous voxels along the shortest path between the peak and the voxel *v*. However it is difficult to know, in the deformed space, which is the shortest path between two voxels. A path composed of 10 voxels may be shorter than a path composed of 6 voxels, if the sum of the distances between the nine pairs of contiguous voxels of the former is smaller than the sum of the distances between the five pairs of contiguous voxels of the latter. A Dijkstra’s algorithm (21) is used in the new method to find the shortest distance between the peak and each of its surrounding voxels. Specifically, the algorithm first calculates the distances between the peak (“initial node”) and each of its’ 26 adjacent voxels. Second, it calculates the distances between one of these adjacent voxels (“current node” in this step) and each its’ adjacent voxels. This step is repeated for each of the voxels adjacent to the peak. Third, it calculates the distances between each of the voxels adjacent to the voxels adjacent to the peak and their adjacent voxels. This is repeated until: (a) the total distance between the peak and a voxel following a path of voxels is not shorter to that previously calculated following another path; or (b) the total distance is longer than the FWHM – which would correspond to the effect size of the peak divided by 16, already negligible.

This algorithm does not restrict its calculations to voxels within a mask of, e.g., gray matter. This is important because neuroimaging studies not uncommonly show significant results outside the expected tissue, due to, e.g., registration mismatches during pre-processing. With this unrestricted spatial propagation: (a) peaks outside the selected mask may be also used in the recreation of the effect size; and (b) researchers can check whether peaks of the recreated map match with those reported in the manuscript – which may be outside the mask. However, spuriously strong correlations outside the tissue might potentially cause artifacts such as “bridges” between two separate brain regions. In order to avoid such artifacts, correlations in voxels with a tissue probability lower than 0.1 in the smoothed average (see Creation of correlation templates below) are decreased proportionally, e.g., are divided by 2 in voxels with a tissue probability of 0.1/2 = 0.05, and by 10 in voxels with a tissue probability of 0.1/10 = 0.01.

### Generalization to variable degrees of anisotropy

Equation 2 can be generalized to:

(5) $$d_{\mathrm{voxel}}=\rho_{\mathrm{isotropic}}^{1-\alpha }\cdot \rho^{\alpha }\cdot d_{\mathrm{peak}}$$

where α is the degree of anisotropy and ρ_isotropic_ is a theoretical spatially constant correlation in the isotropic scenario. Note that α = 0 corresponds to the isotropic scenario, α = 1 to the fully anisotropic scenario, and 0 < α < 1 to variable degrees of anisotropic scenarios.

The theoretical spatially constant correlation in the isotropic scenario (ρ_isotropic_) may be isolated from (3):

(6) $$\rho_{\mathrm{isotropic}}=\exp\left(\frac{-D_{\mathrm{real}}^{2}}{2\cdot \sigma^{2}}\right)$$

where *D*_real_ is the real Euclidean distance between the two voxels.

*D* may be again isolated following the same steps as outlined above:

(7) D=1−α⋅Dreal 2+α⋅2σ2⋅logρ−1

### Subsequent processing steps

Once the deformed distances from a peak have been calculated, these are used by the Gaussian kernel to estimate the effect size of the voxels surrounding a peak. Remaining ES-SDM steps have not been modified: (a) combination of the effect sizes of nearby peaks by means of a weighted average; (b) estimation of the variances associated to these effect sizes; and (c) combination of the effect sizes of the studies included in the meta-analysis by fitting random-effects general linear models. Step (a) is conducted throughout the whole volume for diagnostic purposes, but voxels outside the tissue mask are subsequently discarded.

In ALE, the deformed distances could be used by the Gaussian kernel to estimate the likelihood of a peak, and remaining ALE steps (e.g., the estimation of the probability of the union) would not need to be modified.

## Creation of Correlation Templates

In order to apply the method described above, we needed to create correlation templates for gray matter, white matter, cerebrospinal fluid and fractional anisotropy (FA).

Raw magnetic resonance imaging (MRI) data were obtained from the IXI dataset^1^. This dataset includes nearly 600 MR images from normal, healthy subjects acquired in three different hospitals in London. In order to avoid scanner-related differences, we only used those MR images acquired at Hammersmith Hospital, where a Philips 3 T device was used. T1 parameters were as follows: repetition time = 9.6, echo time = 4.6, 208 phase encoding steps, echo train length = 208, reconstruction diameter = 240, acquisition matrix = 208 × 208, and flip angle = 8. Diffusion tensor imaging (DTI) parameters were as follows: repetition time = 11894, echo time = 51, two averages, 110 phase encoding steps, echo train length = 0, reconstruction diameter = 224, acquisition matrix = 112 × 110, and flip angle = 90.

After exclusion of individuals younger than 20 years or older than 80 years, the Hammersmith Hospital sample included 181 scans. A minimization script was used to select 120 of them in order to obtain 6 equal-sized demographic groups (20–40-year-old males, 40–60-year-old males, 60–80-year-old males, 20–40-year-old females, 40–60-year-old females, and 60–80-year-old females) and a relatively lower frequency of the over-represented white and university-educated individuals (73 and 52% respectively in the original sample, 60 and 40% in the selected sample). The same individuals were used to create the FA template, although DTI data were missing for four of them.

T1 scans were pre-processed following a standard voxel-based morphometry (VBM) algorithm with FSL^2^ , with the exception that no study-specific template was estimated in order that the final images were exactly in Montreal Neurological Institute (MNI) space: brain-extraction (22), tissue segmentation (23), non-linear registration to MNI space, and smoothing (σ = 4mm; FWHM = 9.4 mm). DTI scans were also pre-processed following a standard voxel-based FA algorithm with FSL: brain-extraction, Eddy correction, FA estimation, linear registration to the T1 scans, non-linear registration to MNI space, and smoothing (σ = 3mm; FWHM = 7.1 mm). Non-linear registrations were based on the warp parameters estimated for the T1 gray matter segments.

**Figure 5:**
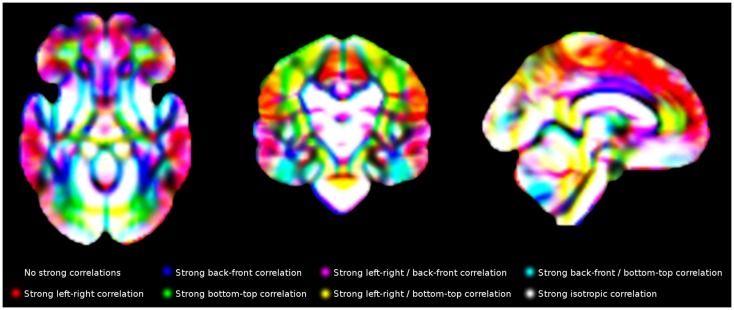
**Main correlation maps for white matter volume**. For illustrative purposes, this Figure only shows correlations along the three main directions (left-right, back-front and bottom-up). The templates created in this study include the correlations with all 26 voxels surrounding each voxel.

Finally, individual values in each voxel were correlated with the individual values in its contiguous voxels using R (24). The gray matter density of a voxel *x* in the 120 individuals, for example, was correlated with the gray matter density of its right-contiguous voxel *y* in the same individuals, with *x* and *y* being variables with one value per subject. A strong correlation would indicate that individuals with more gray matter density in one of the voxels also had more gray matter density in the other voxel. For computational and memory purposes, only 13 correlations were calculated for each voxel, as the other complementary 13 correlation were indeed also calculated for the corresponding neighboring voxel, e.g., “correlation with the voxel at the left” had been already calculated when calculating the “correlation with the voxel at the right” in the voxel at the left. An example of final template is shown in Figure 5.

## Validation of the New Kernel

### Method

In order to validate the new method, six voxel-based effect size maps were recreated from peak information using different degrees of anisotropy and FWHM, and compared to the effect size maps directly obtained from the raw statistical parametric maps (“true” effect size maps). The idea is that the lower the difference between peak-recreated and true effect size maps, the better the recreation.

The 120 individuals from the IXI dataset were divided six times in two groups of 60 individuals each, with these divisions being orthogonal between them (25). A statistical parametric map was obtained from the comparison of the registered and smoothed gray matter segments between the two groups of each pair of groups. The six independent statistical parametric maps were thresholded liberally (*p* = 0.001, with a minimum extent of only 10 voxels) in order to obtain significant differences. The mean (±standard deviation) number of clusters was 22 ± 28, and the median (±absolute deviation) was 16 ± 14.

Effect-size signed differential mapping pre-processing of peak information was then conducted with different degrees of anisotropy (0.0, 0.2… 1.0) and different FWHMs (5, 10… 100 mm). Differences between peak-recreated and true effect size maps were summarized with the relative mean square error (MSE), i.e., the MSE obtained under the current degree of anisotropy and FWHM, divided by the MSE obtained under default ES-SDM isotropic FWHM (20 mm) (6). A relative MSE <100% would indicate an improvement of the recreation. A set of six MSEs (one per statistical map) was obtained for each combination of parameters, and we assessed whether these were lower than the MSEs obtained from the same statistical maps under default ES-SDM isotropic FWHM by means of a non-parametric repeated-measures Wilcoxon signed-rank test.

### Results

**Figure 6:**
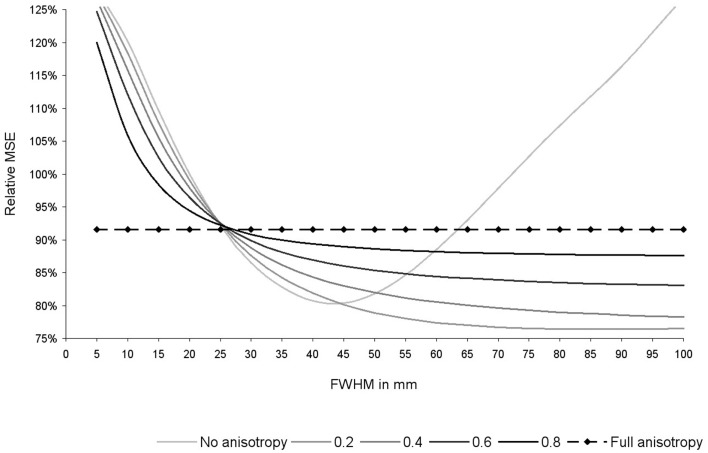
**Relative mean square error (MSE) of the recreation of the statistical maps used in this study depending on the degree of anisotropy and the full-width at half-maximum (FWHM)**. Relative MSE was defined as the MSE obtained with the current set of parameters divided by the MSE obtained after applying effect-size signed differential mapping (ES-SDM) standard isotropic kernel (FWHM = 20 mm). Please note that optimal degree of anisotropy and FWHM were different when using other datasets (not reported here), but use of full anisotropy was still associated to a substantial decrease of MSE.

As shown in Figure 6, the optimal FWHM in this particular dataset ranged from 40–45 mm in the absence of anisotropy (relative MSE = 80%, *p* = 0.053), to 100 mm (or more) when anisotropy was 0.4 or higher (relative MSE = 78–92%, *p* = 0.030–0.053). Narrower FWHMs were associated to substantial increases of the MSE (relative MSE = 120–128%). Wider FWHM also seem to be associated to substantial increases of the MSE, at least in the absence of anisotropy (relative MSE = 127%).

As expected from Eq. 2, the effects of FWHM were null when recreations were conducted with full anisotropy, whereas there was still a substantial decrease of MSE (MSE = 92%, *p* = 0.030).

Optimal degree of anisotropy and FWHM were different when using other datasets (not reported here), but use of full anisotropy was still associated to a substantial decrease of MSE.

## Discussion

This manuscript presents anisotropic kernels for peak-based meta-analytic methods based on the spatial correlation between neighboring voxels, as well as the creation of the required templates for gray matter, white matter, cerebrospinal and FA. The empirical validation showed that the optimal FWHM in the particular dataset used was substantially larger than in previous validations (6, 20), indicating that optimal FWHM might vary largely depending on the data. However, it also showed that the recreation substantially improved and did not depend on the FWHM when full anisotropy was used. Both the method and the templates are readily available with SDM software^3^.

These findings support our hypothesis that isotropic kernels may underestimate the effect size in voxels strongly correlated with the peak (e.g., more likely to be from the same brain region), whereas they may overestimate the effect size in voxels weakly correlated (e.g., less likely to be from the same brain region). In this regard, it must be noted that anisotropic kernels have also already successfully been applied in other neuroimaging fields (26).

In the absence of anisotropy, the optimal FWHM in this validation was found to be 45 mm in the present study, whilst reported to be 20–25 mm in previous work. Such difference might be related to the extent and smoothness of the brain activations, differences or abnormalities. Spatially large and smooth effects may be better recreated with large kernels, whilst small and circumscribed effects with small kernels. Thus, recreation could be optimized for each specific meta-analysis based on the degree of anisotropy and FWHM found to optimally recreate the available statistical maps. This optimization may be achieved following a series of steps analogous to those conducted in Section “Method,” namely: (a) threshold the statistical parametric maps; (b) conduct command-line ES-SDM pre-processing with different degrees of anisotropy and FWHM; (c) calculate the MSE of the differences between each peak-recreated map and the corresponding effect size map under each combination of parameters; (d) choose the optimal parameters based on a plot similar to Figure 6. Robustness should be taken into account when deciding which is the optimal combination of parameters, e.g., ensuring that slight variations in anisotropy or FWHM are not associated with large increase in MSE. On the absence of available statistical parametric maps, however, the use of full anisotropy may represent a robust choice given that results do not depend on the FWHM.

There is at least one situation in which isotropic and anisotropic kernels may be probably equivalent, namely when meta-analyzing studies using tract-based spatial statistics (TBSS) (27). These studies limit their statistical analysis to a FA skeleton, but skeletons of different studies do not overlap. To overcome this difficulty, TBSS protocol in ES-SDM consists in retrieving a mass number of liberally thresholded local peaks from the statistical maps and incorporating them into the ES-SDM TBSS map in order to reconstruct the effect size maps in a common skeleton (28). Given the extreme proximity of the retrieved local peaks, no difference is expected between using one or another kernel. Conversely, the effects of anisotropy may be larger than those found in this paper, in studies reporting few but high peaks, as the shape and intensity of the recreated clusters may differ substantially.

Selecting one or another kernel is obviously irrelevant when the ES-SDM meta-analysis does not include any effect size map recreation from peak information. This could be the case in the rare situations in which statistical parametric maps can be obtained from all the studies in a field. Similarly, this is also the case in the more common situation in which a meta-analytic approach is used to combine data from different sites. This “mega-analytic” approach improves upon simpler covariate-based mega-analyses in that results may be extrapolated to sites other than those included in the multi-site study. Finally, selecting one or another kernel is also irrelevant when combining meta-analytic maps from different modalities (e.g., gray matter volume and BOLD response) to obtain a multi-modal meta-analysis (29–31), as again it does not involve any map recreation from peak information.

Two limitations of this study must be acknowledged. First, the validation showed that the use of full anisotropy may be sub-optimal as compared to some combinations of degree of anisotropy and FWHM. However, full anisotropy is still associated with a significant improvement as compared to default isotropic kernels whilst it is more robust because results do not depend on the FWHM. Second, we did not create a specific correlation template for functional MRI (fMRI) or positron emission tomography (PET). Unfortunately, creation of this template is not straightforward because functional correlations between voxels may depend on the state of mind. The optimal template is likely to be different for each specific fMRI task. Also, we do not know to which extent the functional connectivity abnormalities reported in patients (32) may bias the recreation of the effect size maps of task-based fMRI studies when comparing patients with controls. Fortunately, the use of the structural gray matter template may provide a general correlation template, which seems unlikely to depend on the state of mind or the functional connectivity.

## Conflict of Interest Statement

The authors declare that the research was conducted in the absence of any commercial or financial relationships that could be construed as a potential conflict of interest.

## Footnotes

^1^http://www.brain-development.org/, accessed on Oct 31 2013

^2^http://fsl.fmrib.ox.ac.uk/fsl/fslwiki/

^3^http://www.sdmproject.com
